# Improving Adherence to World Health Organization Guidelines for Pinworm Treatment in Two Primary Healthcare Centers in Misan Province: A Clinical Audit

**DOI:** 10.7759/cureus.93806

**Published:** 2025-10-04

**Authors:** Ahmed Al-Dawoodi, Abbas Al-Elayawi, Samah Hameed

**Affiliations:** 1 Hematology, Misan Hospital for Child and Maternity, Misan, IRQ; 2 The Second Amarah Healthcare Sector, Misan Health Directorate, Misan, IRQ; 3 Public Health, Misan Health Directorate, Misan, IRQ

**Keywords:** clinical audit, enterobiasis, pinworm, soil-transmitted helminths, who guidelines

## Abstract

Background: Soil-transmitted helminths, including enterobiasis (pinworm), remain a significant cause of morbidity, especially among children in moderate and low-resourced countries. Given the noticeable recurrence of this infestation in clinical practice and the distress it causes to children and their families, this audit was carried out to improve adherence to the World Health Organization (WHO) guidelines regarding pinworm treatment and prevention, specifically focusing on correct dosing, repeat dosing intervals, household contact treatment, and hygiene education.

Methodology: A clinical audit of two Plan-Do-Study-Act (PDSA) cycles was conducted in two primary healthcare centers in Iraq. Sixty patients were included in Cycle 1, and 56 patients were enrolled in Cycle 2. Data were collected through a structured questionnaire that focused on anthelmintic dose and repeating interval, treatment of family members, and hygiene advice. The intervention between the two cycles includes the redistribution of enterobiasis treatment and prevention guidelines, as well as the provision of patient leaflets and the use of posters. The statistical analysis was performed using SPSS, in which a chi-square test was utilized to measure the statistical significance in all outcomes except for the age category, where a binomial test was used because the test assumptions for Chi-square were not met.

Results: Following the measures taken, adherence to the recommended dose use showed 8% increase, but this was not statistically significant (*P* = 0.275). The recommended dose interval compliance levelled up from 11% to 37%, treatment of household members elevated to 37% in PDSA Cycle 2, after being 15% in PDSA Cycle 1, and hygiene advice adherence showed a 37% difference between the cycles, which were statistically significant improvements (*P* < 0.05).

Conclusions: This audit demonstrated that adherence to WHO guidelines for pinworm treatment improved after the implementation of several interventions designed to enhance the quality of care. This audit recommends performing similar work in other areas inside and outside Iraq and conducting larger studies focused on the pre-school age group.

## Introduction

Soil-transmitted helminths (STHs) are a worldwide public health issue that causes significant morbidity for adults and children, and their prevalence is closely linked to the socioeconomic status of the populations. The STH include a wide variety of worms, such as roundworm, whipworm, and hookworm infestations [1]. *Enterobius vermicularis*, which is an STH, is the parasite that causes pinworm infestation. The most commonly affected groups in pinworm infestation are children, family members, or those with close contact with an infected person, although anyone can be affected. Pinworm infestation can be transmitted to others by the fecal-oral route, either directly or indirectly. The eggs of this small worm can survive for two to three weeks on surfaces such as clothing, bedding, and household objects [2].

Globally, enterobiasis is a common infestation that affects 12.9% of children, and with the lack of national data in Iraq, we reviewed the prevalence in different countries to get a broader context. In Iran, the prevalence was observed to be 17.2%; in Norway, 18%; and it reached up to 45% in Venezuela, dropping to 2.5% in Colombia. The prevention of this infestation is partially funded by governments, which can be seen as a costly measure. Providing health education to children and their families is a highly successful method to reduce this impact [3-7]. In Iraq, where national data to measure prevalence are absent, a study of 220,607 cases between 2011 and 2015 found that 42.82% of the enterobiasis cases originated from the southern provinces, including 5,696 cases from Misan province [8].

Approximately 43.3% of individuals may remain asymptomatic and might hesitate to seek medical attention due to embarrassment or lack of awareness [9,10]. However, it can also cause various symptoms, such as nocturnal perineal itching, abdominal cramps, insomnia, fatigue, weight loss, irritability, difficulty concentrating, and enuresis, as well as secondary bacterial infections, which can affect development and overall health [11,12].

Enterobiasis in Iraq has a significant effect on the health system, along with its financial burden on the government [8]. Additionally, an extensive systematic review and meta-analysis spanning over 20 years found that overcrowded places, such as schools and kindergartens, as well as inadequate sanitation, are the primary risk factors for this infestation [3]. Enterobiasis responds well to albendazole and mebendazole, which are highly effective alone or in combination, with albendazole being the drug of choice for STH in general [13,14].

In response to the high number of children affected by pinworms in Misan, this audit was conducted to address the issue and support the World Health Organization (WHO) 2030 target of reducing morbidity in pre-school and school-age children, while implementing the Water, Sanitation, and Hygiene (WASH) approach [15].

This clinical audit aims to assess the current practices in two primary healthcare centers (PHCs) in Misan province regarding the recommended anthelmintic dosage, correct timing of repeat dosing, treatment of all household contacts, and provision of hygiene education, and to compare them with the WHO treatment guidelines. This need arises from the recurrent infestations noticed in the PHCs, where the audit was carried out. Additionally, this audit addresses a gap in the literature, as no similar studies have been conducted in this setting previously. Moreover, it aims to promote clinical auditing and evidence-based practice, thereby improving the quality of care, and could serve as an example for conducting audits in low- and middle-resource countries.

## Materials and methods

This clinical audit aimed to assess adherence to the WHO guidelines for the treatment and prevention of pinworm. In Iraq, national guidelines are based on the WHO manual, *Preventive Chemotherapy in Human Helminthiasis*, as shown in Table 1 [16-18]. As per the Iraqi Ministry of Health guidelines, audits do not require formal ethical approval.

**Table 1:** Drugs currently used for the treatment of schistosomiasis, soil-transmitted helminthiasis (STH), and other parasitic infestations. Note: Albendazole and mebendazole should not be given to children under one year of age or to pregnant women during the first three months of pregnancy.

Disease	Drugs	Dose	Frequency of intervention
Enterobiasis (*Enterobius vermicularis*)	Albendazole*	200 mg for children aged 12-23 months; 400 mg for children aged ≥2 years	Repeated after two weeks
	Mebendazole*	100 mg for children aged ≥1 year twice daily for three days	

The audit measured local practice adherence to three standards derived from WHO guidelines. First, all children should receive the anthelmintic dose according to the guideline, and a second dose is given two weeks after the first dose. Second, all household members are supposed to receive treatment, except pregnant women in the first trimester and children less than 12 months of age. Third, all children or their parents should receive personal hygiene advice, especially on regular handwashing with soap and water after bathroom use, diaper changes, and prior to eating, as these actions help prevent autoinfection.

The inclusion criterion was any patient aged one year or older who had previously been treated for pinworm infestation. The exclusion criterion was children diagnosed for the first time, as the audit focused solely on recurrent cases. Because data were collected at the time of presentation, before any treatment, advice, or family member intervention, these children were not eligible for inclusion in the audit, as they had no prior treatment history. Additionally, children under one year were excluded, as guidelines do not recommend medication use in this age group, and children seen at both healthcare centers were included only once.

The audit was conducted in two PHCs, the Second and the Third PHC, which are part of the Second Healthcare Sector in Misan province. The audit was conducted in two (Plan, Do, Study, Act (PDSA)) cycles. The first cycle, referred to as PDSA Cycle 1, was conducted over one month, from May 8, 2022, to June 6, 2022. A sample of 60 patients was consecutively included as they presented to the primary healthcare with recurrent pinworm infestation. The sample included 32 patients from the Second PHC and 28 cases from the Third PHC. A structured questionnaire was developed for data collection, covering age, gender, previous drug use, prescribed dose, the time interval for repeating the second dose, treatment of all household contacts, and personal hygiene advice, without collecting any patient identifiers. Data collection was based primarily on medical records, with the questionnaire completed once the patient was diagnosed with pinworm infestation and before any treatment or advice. The information was then cross-checked with the patient or their parent to ensure accuracy, as shown in the Appendix.

During data analysis and following a discussion with the manager of the infectious disease units and the Second Healthcare Sector manager, we agreed on seven actions to improve adherence to WHO guidelines, preceded by a review of the medical literature. First, the findings were presented along with an action plan in a meeting with the manager of the Second Healthcare Sector and the infectious disease units from all seven PHCs. Second, a copy of the guideline was distributed to all PHCs in Al-Mejar Al-Kabir district to be placed in the workplaces of doctors and pharmacists. Third, a documented notification to the doctors clearly stated that the second dose should be repeated after two weeks, all household members should be treated, and the treating doctors should advise the patients on personal hygiene. Fourth, a patient leaflet explained the definition of pinworm infestation in simple terms, methods of transmission, the treatment, and the importance of treating all household members, as well as providing advice on personal hygiene. This leaflet was given to each patient and their relatives with each prescription. Additionally, it included the date of the second dose to enhance the patient’s commitment to receive the second dose. Fifth, a work group notification summarizing the results and recommendations was sent to the doctors working in the district's PHCs as a reminder. Sixth, a poster explaining the audit results and the action plan was placed in the PHCs where the audit was conducted. Finally, a prevalence study for pinworm infestation in the pre-school age group was recommended, as the group constitutes more than half of the sample, while the national deworming campaign usually focuses on the school-age group, making the pre-school age group an unrecognized group. 

Following these changes, the second cycle of the audit was conducted from December 6, 2022, to January 6, 2023. A sample of 56 patients was selected consecutively as they presented to the primary healthcare in the same way as the first cycle, where 22 cases were from the Second PHC, and 34 cases were from the Third PHC. The same questionnaire form was used for the second cycle to collect the information. Patients in each cycle were divided into three age groups: preschool-aged (<6 years), school-aged (6-15 years), and over 15 years.

Data were analyzed using the IBM SPSS Statistics for Windows, Version 25 (IBM Corp., Armonk, NY), where descriptive statistics were performed to measure percentages, means, standard deviations, and confidence intervals. Furthermore, a chi-square test was used to determine the significance of association for all outcomes except for the age category, where the expected cell count was less than 5. Therefore, a binomial test with a 0.5 cutoff was used to determine if the preschool category, which was the predominant group, represented a statistically significant majority within each cycle. A *P*-value of less than 0.05 was considered statistically significant.

## Results

During PDSA Cycle 1, 60 patients were included after meeting the inclusion criterion. The mean age of the study patients was 5.56 ± 4.56 years, with the youngest child being 1.5 years old and the oldest 35 years old. Male patients comprised 35 (58.3%) of the PDSA Cycle 1 study population. On the other hand, in PDSA Cycle 2, 56 patients participated in the study over one month. The mean age was 5.27 ± 3.35 years, with an age range of 1.5 to 15 years. In contrast to the first cycle, Cycle 2 showed a higher percentage of females, at 33 (58.9%).

It was evident that the majority of patients in both cycles fell within the pre-school age category, accounting for 39 (65%) of patients in PDSA Cycle 1 (*P* = 0.027) and 43 (76%) in PDSA Cycle 2 (*P* < 0.001), as shown in Table 2.

**Table 2:** Patient demographics and age categories. The data are presented as *n* (%).

Cycle	Total patients	Male patients, *n* (%)	Female patients, *n* (%)	Average age (mean ± SD)	Preschool (0-5 years)	School (6-15 years)	Over 15 years
Cycle 1	60	35 (58.3%)	25 (41.7%)	5.56 ± 4.56	39 (65.0%)	20 (33.3%)	1 (1.7%)
Cycle 2	56	23 (41.1%)	33 (58.9%)	5.27 ± 3.35	43 (76.8%)	13 (23.2%)	0 (0%)

In PDSA Cycle 1, regarding the drug dosage, 48 (80%) of the patients received the recommended dose. However, in PDSA Cycle 2, 49 (88%) of 56 patients received the recommended dose of the anthelmintic drug. Although the adherence increased by 8% with a 95% Confidence Interval of -5.8% to 20.8%, this difference was not statistically significant.

**Figure 1:**
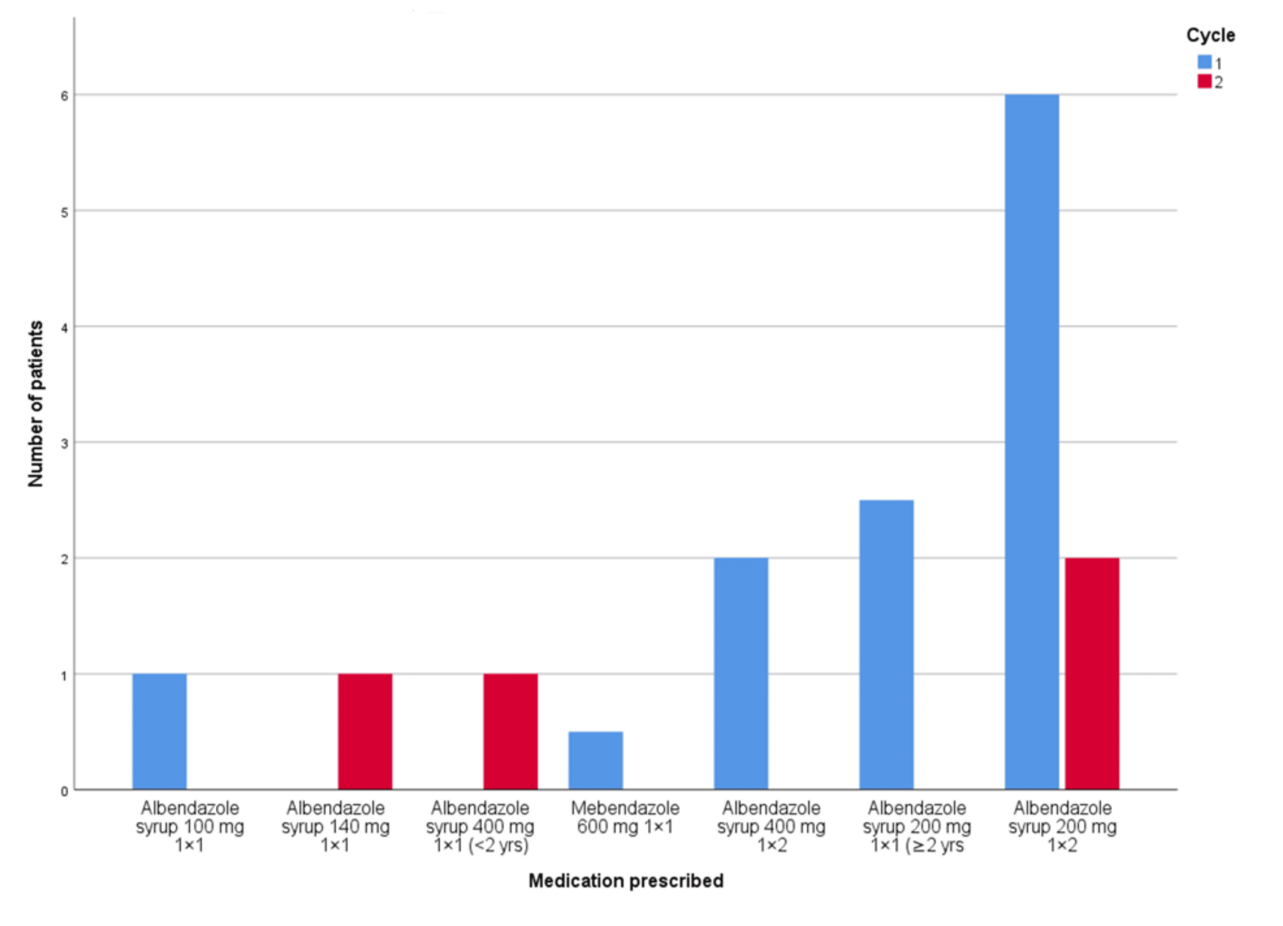
Pattern of non-adherence to the recommended anthelmintic dose.

**Figure 2:**
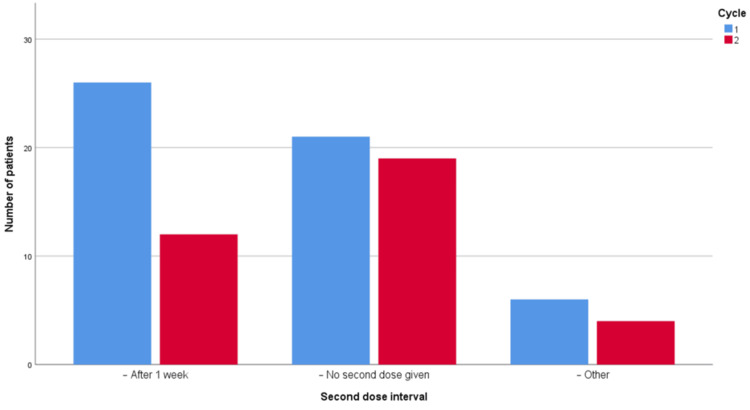
Pattern of non-adherence to the recommended dosing interval.

Regarding the interval for repeating the second dose, anthelmintic treatment was prescribed at the recommended interval in 21 (37%) of patients in PDSA Cycle 2, while only 7 (11%) received the recommended dose in the first cycle. This 26% increase, with a 95% confidence interval of 10.26% to 40.19% is statistically significant. Patterns of non-adherence to the recommended anthelmintic dose and interval are shown in Figures 1-2.

In the context of treating all household members, 21 (37%) of family members in the PDSA Cycle 2 study population received treatment, representing an increase from the first cycle, where only 9 (15%) did. This increase was statistically significant, with a 95% confidence interval for the difference of 6.9% to 38.1%.

Regarding advice on personal hygiene, the analysis indicated that only 24 (40%) patients received this advice in PDSA Cycle 1. In contrast, 38 (67.85%) patients or their parents were advised about personal hygiene in PDSA Cycle 2. This 30% increase was found to be statistically significant, as summarized in Table 3.

**Table 3:** Comparison between the performances of the two audit cycles. χ²: Chi-square statistic *Significant at *P* < 0.001. **Significant at *P* < 0.05.

Variables	Adherence	PDSA Cycle 1 (*n *= 60), *n* (%)	PDSA Cycle 2 (*n *= 56), *n* (%)	*χ*^2^	*P*-value
Recommended dose	Adherent	48 (80%)	49 (88%)	1.19	0.275
	Non-adherent	12 (20%)	7 (12%)		
Recommended dose interval	Adherent	7 (11%)	21 (37%)	10.55	0.001*
	Non-adherent	53 (89%)	35 (63%)		
Households' treatment	Adherent	9 (15%)	21 (37%)	7.64	0.006**
	Non-adherent	51 (85%)	35 (63%)		
Hygiene advice given	Adherent	24 (40%)	38 (67%)	9.034	0.003**
	Non-adherent	36 (60%)	18 (33%)		

Our observations regarding details of non-adherence with the WHO guidance are summarized in Figures 1-2.

## Discussion

This audit aimed to evaluate adherence to WHO recommendations regarding pinworm infestation treatment and prevention in the primary healthcare settings. It revealed measurable improvements in key indicators related to WHO recommendations.

Following the intervention between PDSA Cycle 1 and PDSA Cycle 2, improvements were observed in all indicators, including an increase in adherence to the recommended dose interval from 7 (11%) to 21 (37%) patients, an increase in all household treatment from 9 (15%) to 21 (37%) patients, and a rise in providing hygiene advice from 24 (40%) to 38 (68%) patients. These changes were statistically significant, which may reflect the effectiveness of the measures implemented to improve compliance with the guidelines.

Although pinworm infestation is a common worldwide disease that affects millions of children globally, there is a notable absence of published clinical audits that specifically focus on adherence to management guidelines. Most existing studies are epidemiological, including cross-sectional, retrospective, or meta-analyses that focus on prevalence, risk factors, and outcomes in school or community settings.

In our audit, we evaluated the local practice to identify ways to improve the quality of care and to address the lack of audits that discuss this topic, making this audit novel. We chose this topic due to the recurrence of pinworm infestation that was observed in these PHCs and their impact on the children, their families, the community, and the healthcare system.

Our audit identified the pre-school age category as the most affected age group, with significant public health implications. This finding can be due to the effectiveness of deworming campaigns in schools in Iraq, the difficulty of developing effective hygiene behavior in children less than 6 years old, poor health education in pre-school ages due to the difficulty of accessing these patients until they present to primary care, and fact that children below six are more likely to be exposed to contaminated soil and water as they spend more time outdoors [19-21]. The vulnerability of this age group highlights the significant need to focus on it, as per WHO recommendations, by initiating a preventive chemotherapy campaign similar to the one used in school-age children. This goal aligns with the WHO policy, which has resulted in a 50% increase in the coverage of pre-school children worldwide [22].

Regarding the recommended dose prescription, although the increase in adherence to the recommended anthelmintic dose was not statistically significant, this could be due to high baseline adherence, a small sample size, or the need for a longer timeframe for the audit cycles to be effective. It was also observed that the majority of these inaccurate dose prescriptions are due to prescribing albendazole 200 mg twice daily, which does not provide a therapeutic effect against the parasites [23]. Therefore, setting further interventions and actions, such as intensive education and training for doctors and reviewing prescriptions by pharmacists, may be possible solutions to meet the 100% standard.

Regarding the second dose interval, there was a 26% increase in adherence to the guidelines, indicating the effectiveness of the measures taken. This improvement may be attributed to the reduction in the number of patients who received the second dose after only one week, which was a larger proportion of non-adherence in PDSA Cycle 1. However, there was no significant difference in either cycle regarding the patient who never received a second dose, which became the leading cause of non-compliance in PDSA Cycle 2. This suboptimal adherence could be a contributing factor that may partially explain the challenge of recurrent infestation, as the second dose is proposed to be given in two weeks to kill the hatched ova, which is something that may not be achieved if prescribed after one week or if the second dose is missed, which has been demonstrated in previous studies on STH [17,24]. This omission of the second dose reinforces the need for additional measures, such as contacting parents and establishing a public education campaign.

Regarding the treatment of all household members and personal hygiene, compliance with WHO recommendations increased by 22% and 27%, respectively. Behaviors such as nail biting, inadequate hand hygiene, and low parental literacy have been identified as significant risk factors for pinworm transmission, particularly in rural school-aged populations [25]. These improvements suggest that the sustainability of interventions is required, given the relatively short timeframe between the cycles.

The audit highlighted the importance of adhering to the WHO guidelines when prescribing, providing health education, involving other healthcare professionals such as pharmacists, and conducting further cycles to achieve a long-term effect.

To the best of our knowledge, this is the first published audit in Iraq to compare and evaluate the local practice of pinworm infestation and treatment with WHO guidelines. The audit employed defined, guideline-based criteria to assess the practice, ensuring a reproducible effort. It also used low-cost, clear interventions to ensure a better quality of care is delivered through adherence to the guidelines.

Although it did not lead to complete adherence to the guidance, it has led to significant improvement in clinical practice, which promoted the adoption of its intervention to other primary healthcare centers.

This clinical audit has a limitation in that it includes a relatively small study sample, a short time frame between cycles, a lack of longitudinal follow-up, and the absence of a control group, which can cause immaturity of action plans and thus affect the results. The age group with over 15 years distribution was uneven, with Cycle 1 having one patient and Cycle 2 having no patients, which may represent a minor limitation due to differences between the cycles. Another possible limitation is seasonal variation, as Cycle 1 was conducted in summer and Cycle 2 was conducted in winter. However, a previous study in Iraq has not shown an obvious seasonal variation in pinworm infestation [8], and Cycle 1 was before the school season, and Cycle 2 was after it started.

Despite these limitations, the audit achieved measurable improvement and enhancement in pinworm treatment in primary care.

## Conclusions

This two-cycle clinical audit revealed that several improvements in a short period were likely associated with increasing adherence to WHO guidelines in the treatment and prevention of pinworm infestation. The audit was likely associated with improved compliance from doctors, patients, and relatives regarding the prescription of recommended doses, treating all family members, and adhering to hygiene advice.

The effect of this audit may be maximized by conducting further cycles and implementing further changes to ensure complete adherence to the standards. The audit also recommends performing further cycles to achieve sustainability, replicating similar audits in other PHCs, and conducting larger-scale audits and studies that focus on investigating infestations among pre-school children, both regionally and nationally.

## Figures and Tables

**Figure 3:**
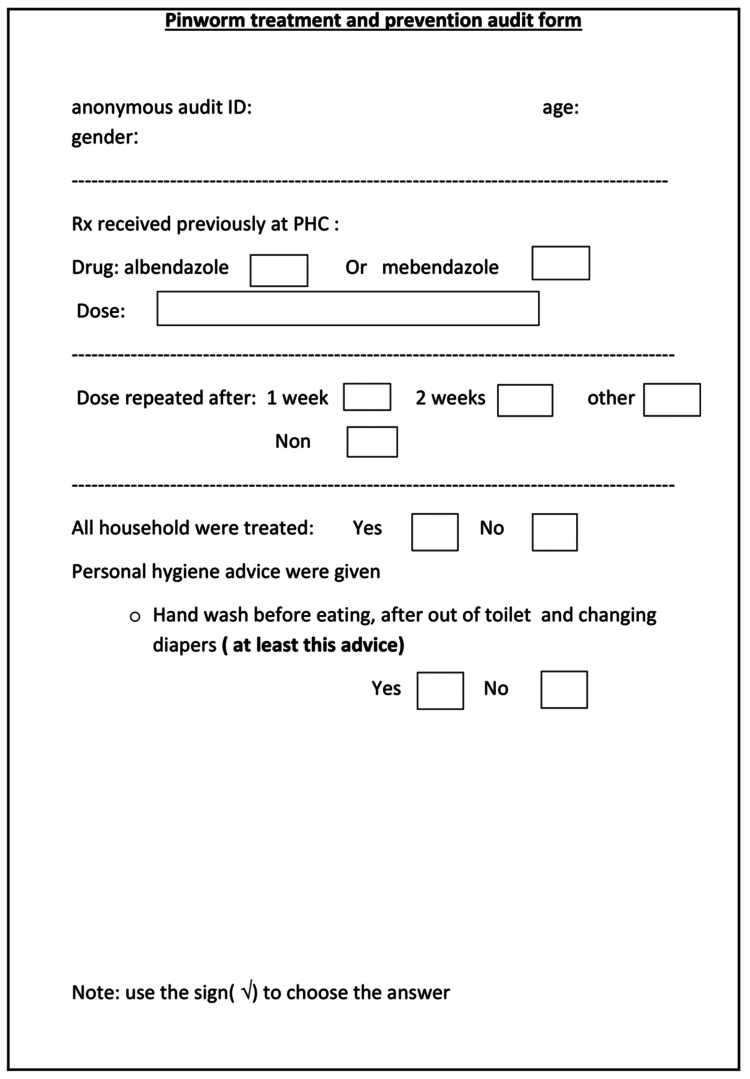
The audit form used for data collection in Cycle 1 and Cycle 2. Credits: Ahmed Al-Dawoodi, Abbas Al-Elayawi, Samah Hameed

## Appendices

Appendix 
